# BDI-Kit: An AI-powered toolkit for biomedical data harmonization

**DOI:** 10.1016/j.patter.2025.101470

**Published:** 2026-02-12

**Authors:** Roque Lopez, Aécio Santos, Christos Koutras, Juliana Freire

**Affiliations:** 1 New York University, New York, NY 11201, USA

**Keywords:** data harmonization, open source, AI agents, large language models, biomedical data, schema matching, value matching

## Abstract

The wide availability of biomedical data, coupled with advanced analytics, holds unprecedented promise for scientific discovery and improved patient care; yet, heterogeneity across datasets remains a major barrier. Given the inherent diversity of biomedical domains, one-size-fits-all solutions are impractical. Despite decades of active research and numerous methods for automating data integration, there is a scarcity of open-source tools capable of handling this complexity. To address these challenges, we introduce Biomedical Data Integration and Harmonization Toolkit (BDI-Kit), an extensible toolkit designed for human-AI collaboration that provides a diverse suite of harmonization methods. It offers two complementary interfaces: a Python API that supports the creation of computational pipelines for harmonization and an AI-assisted chat interface that enables domain experts to perform harmonization using natural language. In this paper, we describe BDI-Kit and demonstrate its capabilities through real-world use cases. By simplifying data harmonization, BDI-Kit empowers researchers and practitioners, facilitating effective exploration and accelerating scientific discovery and clinical research.

## Highlights

• BDI-Kit provides an extensible open-source toolkit for biomedical data harmonization
• Dual interfaces: Python API for data scientists and AI-driven chat for domain experts
• A modular toolkit that integrates multiple schema- and value-matching methods
• Support for human-AI collaboration to improve matching results via iterative refinement

## Teaser

BDI-Kit is an extensible open-source toolkit for harmonizing biomedical datasets. By combining automated methods with AI-assisted guidance, it allows researchers and practitioners to interactively identify and refine how dataset fields and their values correspond across datasets, producing consistent and unified datasets ready for downstream analysis.

## Introduction

In recent years, technological advances have dramatically increased our ability to collect and store vast quantities of biomedical data. These data come from multiple sources, such as routine healthcare visits, clinical trials, and specialized research studies.^1^^,^^2^^,^^3^ The integration of diverse biomedical datasets holds unprecedented promise for accelerating scientific discovery and improving patient outcomes, yet researchers spend a substantial amount of their time preparing and harmonizing data rather than analyzing them and extracting insights. This challenge stems from the fundamental heterogeneity of biomedical data: disparate datasets exhibit differences in their structure (i.e., their schemas) and value formats. While analyzing individual datasets is the first step toward leveraging their information, valuable insights emerge when data are analyzed alongside other relevant datasets. In practice, researchers often integrate related data from different studies, diseases, and patient cohorts to uncover patterns, correlations, and other trends. Furthermore, to facilitate accessibility and sharing, researchers must standardize their data with respect to specific common data models (CDMs).^4^^,^^5^^,^^6^^,^^7^ To automate such processes, data harmonization pipelines need to be assembled that combine methods for integrating and transforming data from multiple data sources into unified and consistent formats and models.

These harmonization pipelines rely on two fundamental tasks: schema matching and value matching. Schema matching focuses on the discovery of relationships between attributes across disparate datasets, typically connecting attributes from a source dataset to the equivalent ones in a target schema. Value matching then operates on identified attribute pairs to find semantically equivalent values between the source and target, often requiring the design of appropriate value transformations. Both tasks have been extensively researched,^8^^,^^9^ resulting in various tools and libraries across different domains.

Various solutions address specific aspects of data harmonization: Trifacta^10^ provides a commercial platform with advanced data-wrangling capabilities for enterprise environments, Valentine^11^ offers a suite of open-source schema-matching methods, and PolyFuzz^12^ is a widely used value-matching library. In the biomedical domain, these tasks are often carried out manually or with the help of tools such as Excel^13^ and Pandas,^14^ while specialized tools, such as BioMart^15^ and TCGAbiolinks,^16^ focus on genomic data integration.

Recent approaches^17^^,^^18^^,^^19^^,^^20^ have proposed the use of large language models (LLMs) to automate a variety of integration tasks, leveraging their broad knowledge and text understanding capabilities. However, they are prone to hallucinations—generating incorrect or nonsensical outputs—and, in the context of harmonization tasks, their capabilities (and computational overhead) may be unnecessary for simple cases, such as when matches differ only in capitalization.

### Challenges in biomedical data harmonization

Biomedical data create unique harmonization challenges, primarily due to the scale and diversity of attributes and values within biomedical schemas. CDMs, such as the Genomic Data Commons (GDC),^4^ contain over 700 distinct attributes, significantly complicating source-to-target schema matching and increasing matching uncertainty. For example, a source dataset column containing country names might correspond to either country_of_residence_at_enrollment or country_of_birth in the GDC schema. Value matching faces a similar challenge: the GDC attribute primary_diagnosis contains over 2,000 possible values, making the identification of a correct source-target value match difficult and error prone. Additionally, harmonization often requires diverse transformation types—textual modifications in some cases and numerical conversions in others (such as converting age from years to days)—demanding flexible, context-aware solutions.

Beyond these inherent matching challenges, significant gaps exist in the available harmonization tools for biomedical applications. First, existing solutions—particularly commercial, closed-source platforms—lack extensibility, preventing users from customizing methods or adding support for new data models as requirements evolve. Second, while automated methods reduce manual effort, they are not foolproof, yet most tools provide limited support for human-in-the-loop refinement, leaving practitioners unable to effectively review and correct errors. Third, the technical expertise required to operate many current tools creates usability barriers for domain experts—clinical researchers and biologists with deep subject-matter knowledge but limited programming experience—thus preventing them from directly participating in the harmonization process that critically depends on their domain expertise.

### Our approach: BDI-Kit

BDI-Kit (Biomedical Data Integration and Harmonization Toolkit) is an open-source toolkit designed to address these limitations through four key capabilities.

(1) Diverse collection of methods for schema and value matching. Rather than relying on a single matching approach, BDI-Kit provides a comprehensive suite of methods, ranging from efficient algorithms to sophisticated AI-powered techniques that can be flexibly combined to handle the scale and semantic complexity characteristic of biomedical schemas. This diversity allows users to balance accuracy and computational costs based on their specific requirements.
(2) Extensibility. BDI-Kit’s modular design enables researchers to contribute new matching algorithms, add support for emerging CDMs, and customize harmonization pipelines. This openness ensures that the toolkit can adapt as biomedical data standards evolve, addressing a critical limitation of closed commercial platforms.
(3) Interfaces for diverse user expertise. BDI-Kit bridges the gap between technical implementation and domain knowledge by offering dual interfaces: a Python API that supports the creation of computational pipelines for developers and an AI-assisted chat interface that enables domain experts to perform harmonization using natural language.
(4) Interactive refinement with human-AI collaboration. Recognizing that automated methods alone cannot guarantee correctness, BDI-Kit integrates AI-assisted suggestions with intuitive mechanisms for human review and correction. This human-in-the-loop design empowers domain experts to iteratively refine matches, combining algorithmic efficiency with human expertise.

In the following sections, we describe the architecture and core functionalities of BDI-Kit. We demonstrate its effectiveness through diverse use cases, ranging from table-to-table harmonization to AI-assisted pipelines, and discuss how, by combining algorithmic diversity, an extensible design, and AI, BDI-Kit provides a comprehensive solution to long-standing challenges in biomedical data harmonization while remaining accessible to both computational experts and domain specialists.

## BDI-Kit

**Figure 1:**
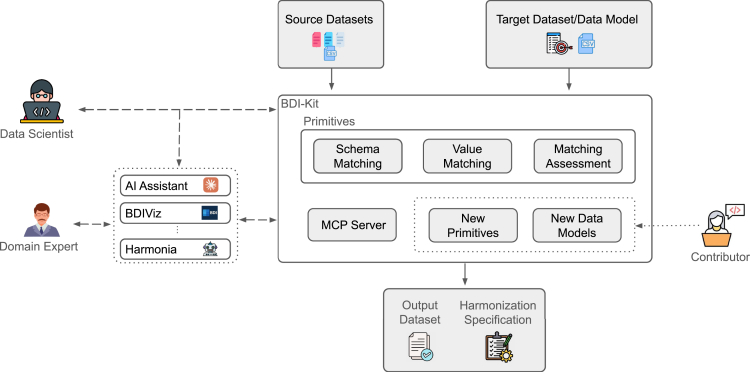
BDI-Kit architecture Given source and target datasets (or common data model), users can construct pipelines that orchestrate schema- and value-matching primitives with human-in-the-loop assessment and refinement of their outputs. Users can interact with BDI-Kit through a Python API or by using AI assistants to generate a harmonized dataset along with a reusable harmonization specification. Contributors can extend BDI-Kit by adding new primitives and support for additional data models.

BDI-Kit is designed to facilitate data harmonization for a diverse range of users, including data scientists and domain experts, while it allows contributors to extend it with additional functionalities and data models, as illustrated in Figure 1. Interaction with the toolkit is achieved either through its Python API or via an AI assistant or agent (e.g., Harmonia^21^).

Users specify the source and target datasets for harmonization. Specifically, users can harmonize any source dataset in tabular format (e.g., a CSV file) to a target dataset, which should also come in tabular format. Additionally, BDI-Kit includes built-in support for widely used data models, such as the GDC,^5^ commonly utilized in cancer research, and Synapse,^22^ frequently employed in studies on neurofibromatosis and related diseases.

To overcome the shortcomings of monolithic solutions, BDI-Kit offers a comprehensive suite of schema- and value-matching algorithms. The toolkit enables refinement of the discovered matches through an iterative process involving humans and AI-powered tools. Moreover, potential contributors can extend BDI-Kit by seamlessly integrating new matching algorithms, additional data models, and target schemas. The final output is the harmonized source dataset, along with a harmonization specification that provides transparency and enables reuse—it can be reused to harmonize different datasets with the same source schema.

BDI-Kit is a Python library. It can be installed via PyPI using the command pip install bdi-kit, and the recently released version, 0.9.0, has been tested on Linux and macOS. The source code for BDI-Kit is available on GitHub (https://github.com/VIDA-NYU/bdi-kit). We have adopted best practices for open-source development, including unit tests, utilizing pytest in conjunction with continuous integration workflows. This process automatically verifies that every new contribution meets the necessary test coverage standards.

### Programmatic API

**Figure 2:**
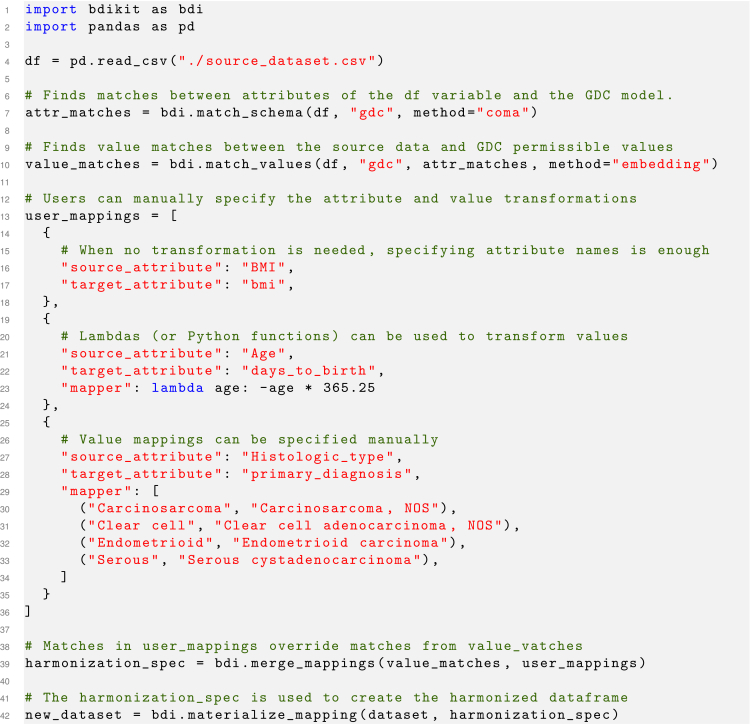
Example of BDI-Kit code snippet in Python The example shows how to use the functions match_schema(), match_values(), and merge_mappings() to create a harmonization specification and generate a new harmonized dataset.

Figure 2 presents a code snippet for a typical data harmonization process, demonstrating how straightforward it is to use BDI-Kit in Python. The process begins with importing the BDI-Kit library (line 1), followed by reading the source dataset (line 4). Next, schema matching is conducted using the match_schema() function. Users can select different matching methods as well as standard target schemas. In this example, we use the Coma schema matcher^23^ and select GDC as the target schema (line 7). The output of match_schema() is a DataFrame that contains one match per row, described by three columns: the *source_attribute* and *target_attribute* attribute names and a *similarity* score quantifying the match’s quality. Subsequently, value matching is performed by invoking the match_values() function, which, similar to match_schema(), supports different methods for value matching. Here, the embedding method is selected (line 10). This function also returns a DataFrame containing values matches for each pair of attribute matches returned by match_schema().

Since fully automated matching algorithms can make mistakes, BDI-Kit provides a mechanism for users to assess and correct matches as well as specify custom transformation functions. This process can be carried out using a domain-specific language (DSL) that allows users to specify a source_attribute, target_attribute, and an attribute mapper for transforming the source attribute into the target format. As shown in lines 13–36, users can provide custom mappings that are merged (using the merge_mappings() function) with the automatically discovered matches using BDI-Kit functions (line 39). When user-provided mappings conflict with automated mappings, user-provided mappings will take precedence. This feature enables users to focus on correcting only the transformations that BDI-Kit did not accurately identify.

The final step involves using the materialize_mapping() function to generate the harmonized dataset (line 42). This function takes the original dataset and the harmonization specification created in the previous step as inputs. It outputs a harmonized DataFrame, which is formatted according to the specification.

### Chat-based interface

**Figure 3:**
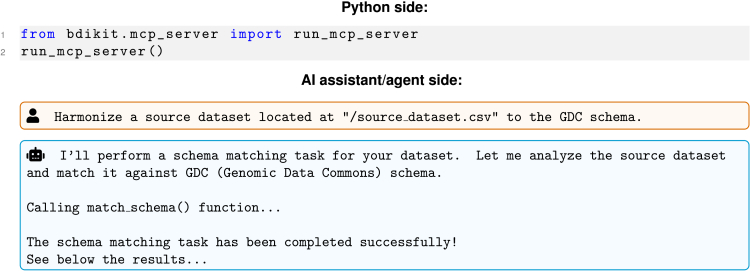
Example of BDI-Kit integration with an AI assistant or agent On the Python side (top), the MCP server is executed, while in the AI assistant or agent side (bottom), users can issue queries and receive answers in natural language.

Figure 3 illustrates how BDI-Kit can be used with an AI assistant or agent. First, the Model Context Protocol (MCP) server must be started; then, users can interact with BDI-Kit through natural language queries issued via their preferred AI assistant or agent. This setup enables a seamless bridge between programmatic functionality and conversational interfaces, allowing users to perform schema matching, value matching, or other BDI-Kit tasks without directly writing code.

For more detailed examples, including Python code and chatbot-style usage, APIs, and advanced usage, refer to our documentation page (https://bdi-kit.readthedocs.io), which provides step-by-step guides that illustrate how to interact with BDI-Kit. In the following, we describe the main features of BDI-Kit.

### Suite of primitives for schema and value matching

To ensure efficiency and flexibility, BDI-Kit integrates a comprehensive set of primitives and algorithms designed to handle different aspects of data harmonization. This includes traditional methods, algorithmic solutions, and LLM-based approaches, ensuring the right tool is available for specific scenarios in schema- and value-matching tasks. These primitives support extracting the top-1 match and can also provide a top-n list of matches for further analysis, enabling users to review and select the most accurate option.

For schema matching, BDI-Kit integrates both widely established methods from the literature, such as Similarity Flooding^24^ and COMA^23^ (originally implemented in the Valentine library^11^), and new methods developed for BDI-Kit, such as Magneto.^19^^,^^25^ Table 1 shows a list of the available schema-matching methods.

**Table 1:** Names and brief descriptions of the schema-matching methods available in BDI-Kit

Name	Description
magneto_zs_bp, magneto_zs_llm, magneto_ft_bp, magneto_ft_llm	Magneto^19^ is a flexible method that can be utilized in multiple configurations; it can use a zero-shot (zs) model or a fine-tuned small language model (ft) for candidate selection, and it can use LLMs (llm) or an efficient bipartite matching algorithm (bp) for enhanced reranking accuracy.
max_val_sim	This schema-matching method has two steps: (1) it first uses a top-k schema matcher supported in BDI-Kit (e.g., magneto_zs_llm) to prune the search space (keeping only the top-k most likely matches) and then (2) it uses a value matcher method to rerank the top-k candidate matches, taking into account how well their values match the values from the source column.
two_phase	The two_phase schema-matching method first uses a top-k schema matcher (e.g., magneto_zs_llm) to prune the search space (keeping only the top-k most likely matches) and then uses another schema matcher to choose the best match from the pruned search space.
llm	Leverages LLMs to identify and select the most accurate schema matches; supports multiple models, with gpt-4o-mini used as the default.
similarity_flooding	Similarity flooding^24^ transforms schemas into directed graphs and merges them into a propagation graph; the algorithm iteratively propagates similarity scores to neighboring nodes until convergence.
coma	COMA^23^ is a matcher that combines multiple schema-based matchers, utilizing a multitude of string and set similarity metrics.
cupid	Cupid^26^ is a schema-based approach that translates schemas into tree structures; it calculates overall similarity using linguistic and structural similarities, with tree transformations helping to compute context-based similarity.
distribution_based	Distribution-based matching^27^ compares the distribution of data values in columns using the Earth mover’s distance; it clusters relational attributes based on these comparisons.
jaccard_distance	This algorithm, described in Koutras et al.,^11^ computes pairwise column similarities using Jaccard similarity, treating values as identical if their Levenshtein distance is below a threshold.

Similarly, for value matching, BDI-Kit offers a variety of approaches. These include text-based transformations, such as term frequency-inverse document frequency (TF-IDF), and distance-based methods, as well as semantic transformations using embedding-based methods from pre-trained models, such as the BioBERT model.^28^ Additionally, it supports numeric conversion methods (e.g., transforming the age expressed in years to days). BDI-Kit also integrates LLM-based methods, enabling it to handle more complex value-matching tasks. Table 2 presents a detailed list of the available value-matching methods.

**Table 2:** Names and brief descriptions of the value-matching methods available in BDI-Kit

Name	Description
edit_distance	Uses the edit distance between lists of strings using a customizable scorer that supports various distance and similarity metrics
tfidf	This method leverages the term frequency-inverse document frequency (TF-IDF) weighting to quantify the similarity between strings based on their shared n-gram features
embedding	A value-matching algorithm that leverages the cosine similarity of value embeddings for precise comparisons; by default, it utilizes the bert-base-multilingual-cased model to generate contextualized embeddings, enabling effective multilingual matching
llm	Leverages LLMs to identify and select the most accurate value matches; supports multiple models, with gpt-4o-mini used as the default
llm_numeric	Employs LLMs to perform numeric value transformations, such as converting ages from years to months; supports multiple models, with gpt-4o-mini used as the default

The LLM-based primitives for both schema and value matching are model agnostic, allowing users to specify the desired LLM as an additional parameter (e.g., match_values(dataset, method = "llm", method_args = {"model_name": "ollama/gemma2:27b"})). This flexibility supports the use of either open-source or commercial models, which can encourage the adoption of the tool across diverse user communities. Moreover, users working with sensitive data can securely choose an open-source LLM option, avoiding the need to transmit data to external commercial services.

BDI-Kit also offers primitives to evaluate and explain the matches produced by its algorithms. These methods return one of three possible labels, accept, needs review, or reject, accompanied by a brief explanation detailing the rationale behind the decision. They provide insights into why the algorithm matched a specific pair of attributes or values and whether the match is valid, invalid, or requires further consideration. By leveraging these primitives, users can actively participate in improving the matching process by identifying and addressing potential errors made by the automated matching algorithms.

### Harmonization specification and interactive refinement

In BDI-Kit, the schema- and value-matching functions are composable, i.e., their outputs can be used as input to another function to create a data harmonization pipeline. These outputs can also be combined with matches provided by users (humans or AI-powered tools) to compose a declarative harmonization specification that fully specifies how to transform a source dataset into a harmonized dataset. Besides providing transparency and supporting reproducibility for the harmonization process, the specification can be reused in future tasks to harmonize new datasets.

**Figure 4:**
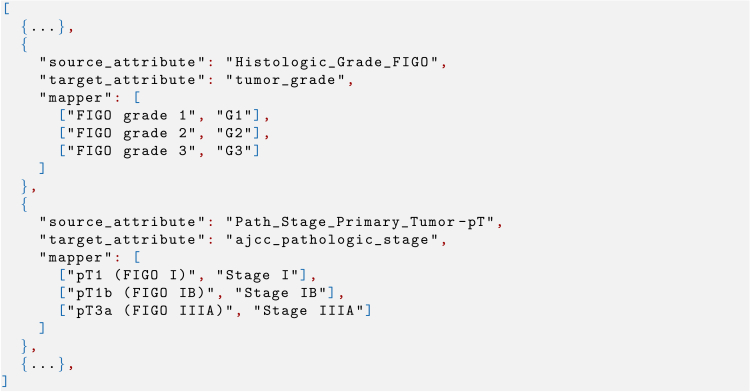
A snippet from the harmonization specification The example shows the harmonization specification for the attributes Histologic_Grade_FIGO and Path_Stage_Primary_Tumor.

Figure 4 shows a snippet of a generated harmonization specification, which provides a clear, declarative representation of the schema and value matches in JSON format. Each entry defines how specific source attributes and their corresponding values should be transformed to align with the target schema. In this example, the snippet focuses on dictionary-based transformations; however, BDI-Kit also supports other types of transformations, such as user-defined Python functions.

User input is essential when matching ambiguous attributes. For example, when finding matches for an attribute named Path_Stage_Primary_Tumor-pT, a user might want to manually choose whether it should be aligned with ajcc_pathologic_t or uicc_pathologic_t. Users can also define custom mapping functions to address cases where the default algorithms may be ineffective or when a mathematical function is required to transform values (as in lines 19–24 of Figure 2). These custom functions can be passed as arguments to create the harmonization specification, allowing them to be easily integrated into the data transformation pipeline.

This process of progressive refinement of the harmonization specification, done by reviewing uncertain matches and making corrections as needed, is interactive and driven by the users. To support this process, BDI-Kit provides confidence scores for each match, which can be used to identify and flag potentially inaccurate matches.

### Extending the toolkit with primitives and data models

BDI-Kit is built with an open, extensible architecture, allowing the broader community to contribute new primitives, algorithms, and data models. This design fosters collaboration among researchers, developers, and domain experts, creating a dynamic and evolving toolkit that can effectively handle the challenges of biomedical data harmonization. Users can add custom matching algorithms to address specific biomedical data types or incorporate new data models, such as Gene Expression Omnibus (GEO),^29^ ensuring BDI-Kit remains versatile and relevant to new research demands.

To simplify the integration of other schema- and value-matching solutions, contributors need to implement dedicated methods. For example, a schema-matching primitive can be easily integrated with BDI-Kit by implementing the match_schema() function, whereas match_value() can be used instead for integrating value-matching solutions. Similarly, to add support for a new data model, contributors need to extend the BaseStandard class and implement four methods: get_attributes(), get_attribute_values(), get_attribute_metadata(), and get_dataframe_rep(). The storage format of the data model is flexible: it can be JSON, YAML, or any other format, since all modules in BDI-Kit interact with the data model exclusively through these four methods to retrieve the necessary information. Once implemented, these contributions are fully incorporated into BDI-Kit’s workflow. Contribution guidelines and step-by-step technical details for adding new primitives and data models are available on our documentation page (see the “contributing” section).

This extensibility allows users to tackle specialized challenges, customize the toolkit to their needs, and future-proof it for emerging biomedical data models. For example, a user working with gene sequencing data can design a tailored matching algorithm for gene alignment or create a new matcher for harmonizing patient demographics across datasets. Contributions are integrated into the main project via a straightforward process of opening a *pull request* for approval, ensuring quality and consistency across updates.

### Enhancing accessibility through AI-powered tools

AI assistants and agents have demonstrated remarkable performance in conversational workflows, providing intuitive and efficient ways for users to interact with complex systems. Recognizing their potential, BDI-Kit has been designed for seamless access through AI-powered tools, allowing users to perform advanced data harmonization tasks using natural language commands. In previous work, we developed Harmonia,^21^ a system that integrates BDI-Kit functions with a computational notebook environment, allowing users to interleave Python code with commands written in natural language. While this was a promising approach, the system was restricted to the Beaker user interface^30^ and relied on OpenAI’s commercial GPT-4o model.

Over the past year, with the introduction of the MCP,^31^ we have witnessed a surge of AI assistants that can use tools that implement this protocol. To improve compatibility with these tools and encourage broader adoption of BDI-Kit, we developed an MCP server that encapsulates the core functions of BDI-Kit. This MCP server acts as a bridge between BDI-Kit and AI assistants, enabling seamless communication using the MCP protocol. As a result, BDI-Kit’s primitives and algorithms are now accessible in real time, allowing users to interact with the toolkit through the AI assistants and models they prefer.

For instance, users can simply say “find the best match for these columns” or “transform the values of the column tumor_focality to align to GDC,” and the AI-powered tool processes the request through the primitives implemented in BD-Kit. Additionally, agents can serve as reasoning mechanisms; for example, they can select appropriate primitives to be used or correct potential errors. This approach not only lowers the usability barrier for non-technical users but also enhances productivity by streamlining complex harmonization tasks.

## Use cases

### Table-to-table harmonization

Generating molecular biomedical data is costly, but reanalyzing public omics data alongside one’s own dataset is increasingly feasible with the growth of public repositories. However, this presents challenges, as multiple standards are applied across datasets. Clinical variables are often named differently, and their values are not necessarily standardized, making manual integration tedious and time-consuming. Consider the following use case, where a biomedical researcher seeks to integrate clinical data tables from two published cohorts of proteogenomics data for endometrial tumors from the CPTAC consortium.^32^^,^^33^

The researcher begins by loading both tables from CSV files. BDI-Kit provides a suite of functions designed to identify matches between attributes/columns in the source and target datasets. To accomplish this with BDI-Kit’s Python API, the researcher uses the function call match_schema(source = df_dou_2020, target = df_dou_2023, method = "magneto_zs_llm"), where df_dou_2020 and df_dou_2023 are the source and target datasets loaded as Pandas DataFrames, respectively, and "magneto_zs_llm" specifies the schema-matching method.

After identifying matching attributes, the next step is to standardize the column values. BDI-Kit facilitates this process with its match_values() function, which identifies values in the source dataset that should align with those in the target dataset. The library supports various methods for this task, including both syntactic and semantic matching algorithms. In this use case, the researcher employs the tfidf method, which determines matches based on the similarity of character n-grams, effectively identifying closely related values.

**Figure 5:**
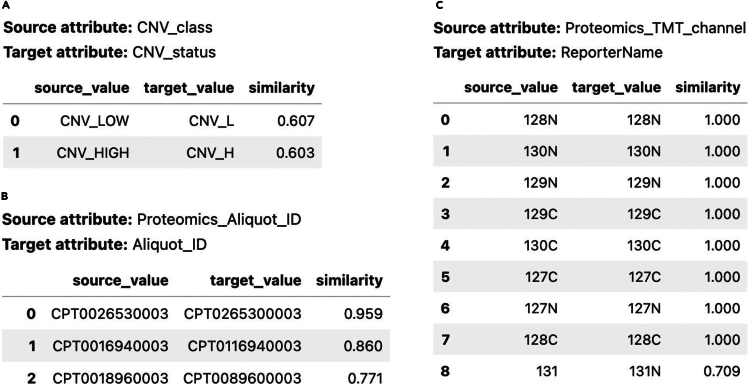
Results of calling match_schema() and match_values() functions (A) Attributes and their corresponding values were correctly aligned despite spelling variations. (B) Precise matching of identifiers. (C) Accurate attribute matching even when values contain typos.

In Figure 5, we present sample results obtained by invoking both functions—for each matched attribute, we can see its associated matched values. In Figure 5A, the attributes CNV_class → CNV_status were successfully matched. Additionally, the corresponding values were accurately matched. For example, the pairs CNV_LOW → CNV_L and CNV_HIGH → CNV_H were correctly aligned despite differences in their spelling. Similarly, in the attributes Proteomics_Aliquot_ID → Aliquot_ID, the aliquot IDs were matched with high precision (Figure 5B). In the case of the matched attributes Proteomics_TMT_channel → ReportName (Figure 5C), the value 131 was mapped to 131N, suggesting a potential typo detected by the matching algorithm. This demonstrates the ability of BDI-Kit to handle subtle variations and ensure reliable integration.

After verifying and refining the value mappings, the researcher proceeds to generate a harmonized dataset. This can be achieved in BDI-Kit using the materialize_mapping() function, which applies the finalized mappings to produce a unified table ready for further analysis.

### Table-to-model harmonization

BDI-Kit also supports the harmonization of a given dataset to a standard schema. This use case demonstrates how an input dataset formatted according to the GEO data model is harmonized with the Synapse data model to ensure compatibility for upload to the Synapse platform.^22^ Notably, not all attributes in the source dataset need to be mapped to the target schema.

The data scientist addresses this challenge by first obtaining the top match suggestions and then filtering them as needed. The max_val_sim method is chosen due to overlapping attributes between the source dataset and the target data model.

**Figure 6:**
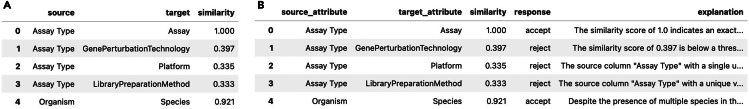
Sample results of attribute matches with their explanations (A) Sample of top-ranked schema matches retrieved for *Assay* and *Organism* attributes. (B) Evaluation of these matches, highlighting accepted and rejected matches.

The rank_schema_matches(df_source_data, "synapse", top_k = 4, "max_val_sim") function is called, where df_source_data represents the source data, "synapse" specifies the target data model, and the top 4 matches are retrieved using the "max_val_sim" method. The results of this call are shown in Figure 6A. Following this, the user invokes the evaluate_schema_matches() method to analyze these matches (see Figure 6B), identifying potential reasons for considering them as accepted or rejected. This step also helps determine whether further review is required for some matches.

For instance, the matches Assay Type → Assay, and Organism → Species are considered as valid matches. In the first case, as determined by BDI-Kit, the main reason for considering this an accepted match is that their unique values, RNA-Seq and RNA-seq, respectively, refer to the same assay method in the context of biological assays. In contrast, the match Assay Type → LibraryPreparationMethod is rejected because the source attribute directly points to a type of sequencing method, while the target attribute lists various library preparation kits and methods, some of which are related to sequencing but are not sequencing methods themselves.

### Using AI agents to interact with BDI-Kit

In the previous use cases, we demonstrated how BDI-Kit can be utilized by users with programming experience. In this use case, we illustrate how domain experts, even with limited programming expertise, can leverage BDI-Kit through an AI agent to harmonize biomedical data. This process emphasizes an interactive workflow where the AI agent, powered by BDI-Kit’s function-calling capabilities, guides schema- and value-matching tasks. For this demonstration, we used a Streamlit application (app) that allows users to issue queries in natural language. Internally, the app uses the open-source LLM gpt-oss-120b, although other models can also be specified as parameters. Detailed instructions for configuring this app are provided in the examples included in our repository.

**Figure 7:**
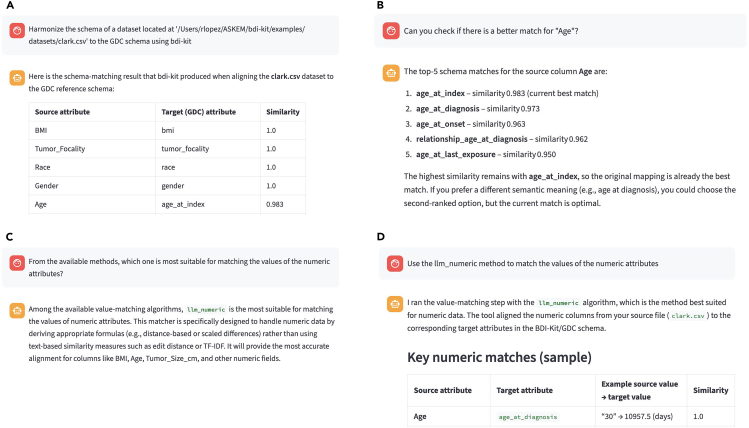
BDI-Kit accessed directly from an AI agent (A) Initial schema matches are generated in response to the user’s request. (B) Alternative matches for the Age attribute obtained using the rank_schema_matches() function. (C) Selection of the method for value matching in numeric attributes. (D) Example of the transformation applied to convert Age values.

The process begins with a user request to harmonize a dataset related to Renal Cell Carcinoma^34^ to the GDC standard, as shown in Figure 7A. The AI agent initiates the process by calling BDI-Kit’s match_schema() function. The initial results reveal a perfect alignment for many columns, e.g., BMI → bmi, Tumor_Focality → tumor_focality, Race → race, and Gender → gender.

While most matches are accurate, the user expresses skepticism about the match Age → age_at_index, which has a slightly lower confidence score (98.3%), and asks, “Can you check if there is a better match for Age?” In response, the AI agent utilizes BDI-Kit’s rank_schema_matches() function to explore additional alternatives. Guided by semantic understanding, the agent identifies age_at_diagnosis as a potential match (see Figure 7B).

After updating this match and with the schema largely aligned, the user proceeds to the value-matching step, asking, “From the available methods, which one is most suitable for matching the values of the numeric attributes?” As shown in Figure 7C, the AI agent queries BDI-Kit for available value-matching algorithms and recommends using the llm_numeric method, which is specifically designed for numerical data. Finally, the agent identifies the corresponding value matches for this data type (Figure 7D), providing an example and explanation of the transformation, from values expressed in years (the Age attribute) to values expressed in days (the age_at_diagnosis attribute).

This use case demonstrates a powerful and flexible data harmonization process enabled by the integration of BDI-Kit with AI-powered tools. The knowledge embedded in LLMs can guide the selection of better candidates in cases where BDI-Kit does not identify the optimal match and can also assist in interpreting the performed transformations. Moreover, the conversational interface supports an intuitive and iterative process for both schema and value matching.

We also ran this use case using commercial AI assistants. Specifically, we executed BDI-Kit from the Claude desktop app. Compared to the open-source LLM agents, Claude required no additional context prompts to correctly interpret the task and interact with BDI-Kit. In contrast, some smaller open-source models, such as deepseek-r1:32b, even with detailed instruction prompts, occasionally failed to pass the correct function arguments to BDI-Kit, leading to execution errors or incomplete requests.

## Discussion

### Limitations

Although BDI-Kit has been successfully applied in several use cases, it has limitations. In terms of supported matching types, it currently handles one-to-one and one-to-many mappings, where a source attribute is matched to a single or multiple attributes in the target schema, respectively. Specifically, one-to-many mappings are possible since BDI-Kit can provide the top-k candidate matches for a source attribute (e.g., *hispanic* may be mapped to both *Hispanic-Latino* and *Spanish Origin*). However, BDI-Kit does not currently support many-to-one mappings. For example, if we have the attributes *weight* and *height*, they could be combined into a single target attribute, such as *BMI*, since this value results from merging both attributes.

The input dataset for BDI-Kit must be provided as a single Pandas DataFrame. We chose this format because it is widely used for performing operations and analyses on datasets, and it supports loading data from multiple file formats (e.g., CSV, Excel, and Parquet). However, datasets distributed across multiple files are not directly supported in BDI-Kit and must first be merged into a single file. Tools such as Pandas^14^ provide functions for transforming and merging datasets, which BDI-Kit can readily leverage. BDI-Kit also does not provide data-cleaning primitives (e.g., imputation). Although it supports missing values and different types of values (e.g., text, categorical, numeric, and date-time), it assumes that the dataset has already been cleaned during a prior preprocessing step. Since the toolkit is written in Python, users can combine BDI-Kit with other libraries that support data cleaning.

Another limitation concerns the matches produced by BDI-Kit. As mentioned earlier, the matches returned by its primitives are not always correct, which is why BDI-Kit supports user refinements to adjust the results when necessary. Even when BDI-Kit is used through AI assistants or agents, errors may still occur, particularly in complex cases (e.g., when the target schema contains a large number of attributes). Therefore, user review remains an essential part of the process. Tools such as BDI-Viz,^35^ a visual analytics system that uses BDI-Kit in its backend, can assist in reviewing the results of schema-matching tasks.

Finally, although there is an initial learning curve to understanding BDI-Kit’s functions and the format for user refinements, we posit that this investment is justified by the benefits. BDI-Kit significantly reduces the manual work required for schema and value matching, improves the reproducibility of the process, and scales as the number of datasets and attributes increases.

### Future work

There are several directions we plan to pursue in future work to address some of the limitations and enhance BDI-Kit’s capabilities. We aim to support more complex scenarios, such as many-to-one matches, which involve consolidating multiple attributes or values into a single target. Supporting such matches will require extending the current matching primitives to reason over attribute dependencies and transformations, as well as incorporating semantic knowledge where necessary. To further improve usability, we plan to work on integrating an AutoML-based approach^36^ that can automatically recommend the most suitable schema- and value-matching algorithms for a given dataset, taking into account factors such as data type, size, and complexity. This will reduce the need for manual configuration and allow users to achieve optimal harmonization results more efficiently. Additionally, we intend to implement learning mechanisms, such as reinforcement learning or active learning, enabling BDI-Kit to learn from user feedback and past performance. Over time, this approach will allow the toolkit to refine its algorithm selection, improve match accuracy, and better handle diverse datasets with minimal supervision.

## Resource availability

### Lead contact

Requests for further information and resources should be directed to and will be fulfilled by the lead contact, Juliana Freire (juliana.freire@nyu.edu).

### Materials availability

The BDI-Kit library is hosted on PyPI and can be accessed at https://pypi.org/project/bdi-kit/.

### Data and code availability

The source code for BDI-Kit is publicly available on GitHub at https://github.com/VIDA-NYU/bdi-kit. The specific version used in this paper (0.9.0) is archived on Zenodo at https://doi.org/10.5281/zenodo.17634387.^37^ It has been released under the Apache 2.0 license. The datasets for use cases 1 and 3 are included in the repository. However, the dataset for use case 2 cannot be released due to privacy restrictions. It can be accessed through Synapse at https://www.synapse.org/Synapse:syn62827833 but requires submitting an access request via the Sage Bionetworks support portal at https://sagebionetworks.jira.com/servicedesk/customer/portals.

## Acknowledgments

This work was supported by the 10.13039/100000185DARPA ASKEM program agreement no. HR0011262087 and the ARPA-H BDF program. The views, opinions, and findings expressed are those of the authors and should not be interpreted as representing the official views or policies of DARPA or ARPA-H. We thank Eduardo H.M. Pena and Eden Wu for their feedback and contributions to the code. We also thank the anonymous reviewers for their insightful comments and suggestions, which we used to improve both the manuscript and BDI-Kit.

## Author contributions

Conceptualization, R.L., A.S., and J.F.; methodology, R.L., A.S., and J.F.; investigation, R.L., A.S., C.K., and J.F.; writing – original draft, R.L. and C.K.; writing – review & editing, R.L., A.S., C.K., and J.F.; funding acquisition, J.F.; resources, R.L., A.S., and J.F.; supervision, C.K. and J.F.

## Declaration of interests

The authors declare no competing interests.

## Declaration of generative AI and AI-assisted technologies in the writing process

During the preparation of this work, the authors used ChatGPT and Claude to enhance the clarity and flow of the text. After using this tool or service, the authors reviewed and edited the content as needed and take full responsibility for the content of the publication.
